# Co-activator independent differences in how the metaphase and anaphase APC/C recognise the same substrate

**DOI:** 10.1242/bio.20149415

**Published:** 2014-09-12

**Authors:** Takahiro Matsusaka, Maria Enquist-Newman, David O. Morgan, Jonathon Pines

**Affiliations:** 1 The Gurdon Institute, Tennis Court Road, Cambridge CB2 1QN, UK; 2 Department of Zoology, University of Cambridge, Tennis Court Road, Cambridge CB2 1QN, UK; 3 Department of Physiology, University of California in San Francisco (UCSF), 600 16th Street, San Francisco, CA 94158, USA

**Keywords:** Mitosis, Anaphase Promoting Complex/Cyclosome, Cyclin B

## Abstract

The Anaphase Promoting Complex or Cyclosome (APC/C) is critical to the control of mitosis. The APC/C is an ubiquitin ligase that targets specific mitotic regulators for proteolysis at distinct times in mitosis, but how this is achieved is not well understood. We have addressed this question by determining whether the same substrate, cyclin B1, is recognised in the same way by the APC/C at different times in mitosis. Unexpectedly, we find that distinct but overlapping motifs in cyclin B1 are recognised by the APC/C in metaphase compared with anaphase, and this does not depend on the exchange of Cdc20 for Cdh1. Thus, changes in APC/C substrate specificity in mitosis can potentially be conferred by altering interaction sites in addition to exchanging Cdc20 for Cdh1.

## INTRODUCTION

The cell cycle is driven by alternating states of high and low cyclin-dependent kinase activity (Morgan, 2007). Ubiquitin-mediated proteolysis underpins this by destabilising specific cyclins at particular times, which is especially important for the B-type cyclins that drive the cell into mitosis (Murray et al., 1989; Wolf et al., 2006). B-type cyclins are targeted for destruction only when the Spindle Assembly Checkpoint (SAC) is inactivated when all the chromosomes have properly attached to the mitotic spindle (Clute and Pines, 1999). The B-type cyclins continue to be degraded through the next G1 phase until cells have re-licensed chromosomes for the next round of DNA replication (Amon et al., 1994; Brandeis and Hunt, 1996); hence each cell division must normally be followed by a round of DNA replication.

The B-type cyclins are ubiquitylated (Glotzer et al., 1991) by the Anaphase Promoting Complex (King et al., 1995; Yamashita et al., 1996; Zachariae et al., 1996) or Cyclosome (Sudakin et al., 1995) (APC/C), a multi-subunit ubiquitin ligase that recognises a variety of different proteins in mitosis and in G1 phase (reviewed by Barford, 2011; Pines, 2011). The APC/C is able to select different substrates at different times in mitosis (Pines, 2006), but how it does so is still unresolved. In early mitosis the APC/C is activated by the Cdc20 protein that itself is regulated by the SAC (Fang et al., 1998; Hwang et al., 1998; Kim et al., 1998; Sudakin et al., 2001; Yu, 2007). The SAC detects improperly attached chromosomes (Hoyt et al., 1991; Li and Murray, 1991; Murray, 2011; Rieder et al., 1995) and inactivates Cdc20 to prevent the APC/C from recognising securin and cyclin B1, thereby preventing sister chromatid separation and exit from mitosis, respectively. While the SAC is active, however, the APC/C can still recognise some of its other substrates, notably cyclin A (Di Fiore and Pines, 2010; den Elzen and Pines, 2001; Geley et al., 2001; Wolthuis et al., 2008) and Nek2A (Hayes et al., 2006). Only when the SAC is inactivated in metaphase does the APC/C begin to degrade securin and cyclin B1 (Clute and Pines, 1999; Hagting et al., 2002). Part of the explanation for why some substrates, such as cyclin A and Nek2A, are degraded earlier than others in mitosis is because they bind directly to the APC/C (Di Fiore and Pines, 2010; Hayes et al., 2006; Wolthuis et al., 2008). But recruitment alone is not sufficient to confer earlier degradation because neither Kif18A nor cyclin B1 are degraded while the SAC is active despite being bound to the APC/C (Sedgwick et al., 2013; van Zon et al., 2010). Thus, in addition to binding to the APC/C, cyclin A also recruits Cdc20 (Di Fiore and Pines, 2010; Wolthuis et al., 2008), and Nek2A can be degraded *in trans* when the amino-terminus of Cdc20 or the related protein, Cdh1 are added to activate the APC/C (Kimata et al., 2008).

Once cells begin anaphase the APC/C recognises a wider variety of substrates, including Plk1, the Aurora kinases, and Cdc20 itself (Floyd et al., 2008; Lindon and Pines, 2004; Littlepage and Ruderman, 2002; Pfleger et al., 2001). The anaphase and G1 phase APC/C ubiquitylate a broader range of substrates and a number of APC/C degrons have been identified in addition to the classical ‘Destruction box’ (consensus: RxxLxxI/VxN) that mediates Cyclin B1 and securin destruction (Glotzer et al., 1991; King et al., 1996; Yamano et al., 1998; Zur and Brandeis, 2001) (note that lysine substitutes for arginine in Drosophila securin (Leismann et al., 2000)). These degrons include the KEN box (Pfleger et al., 2001) (consensus: KENxxxN/D), and other motifs such as the O-box (Araki et al., 2003) or the GxEN motif (Castro et al., 2003) that often resemble degenerate D-boxes or KEN-boxes (Barford, 2011). The molecular mechanism behind this change in substrate specificity is partially attributable to the replacement of Cdc20 by Cdh1 (Visintin et al., 1997; Zur and Brandeis, 2002). For example, Aurora A is degraded in anaphase but only in cells with Cdh1 (Floyd et al., 2008; García-Higuera et al., 2008; Sigl et al., 2009), and the D-box of Hsl1 is recognised by APC/C bound to Cdc20 whereas its KEN-box is recognised by APC/C bound to Cdh1 (Burton and Solomon, 2001).

The change in the substrate specificity of the APC/C in anaphase is not, however, as simple as a switch from D-boxes to KEN-boxes when Cdc20 is replaced by Cdh1. For example, both Plk1 and Aurora A are degraded in anaphase but this requires a D-box (Lindon and Pines, 2004; Littlepage and Ruderman, 2002) rather than a KEN box (Littlepage and Ruderman, 2002) (plus an A-box in the case of Aurora A (Littlepage and Ruderman, 2002)). It has been suggested that Plk1 is degraded before Aurora A in anaphase simply because it is a more processive substrate (Rape et al., 2006), but there appears to be a more qualitative difference because Aurora A can only be recognised by the APC/C bound to Cdh1 (Floyd et al., 2008; García-Higuera et al., 2008; Sigl et al., 2009) whereas Plk1 can be degraded in anaphase by the APC/C whether or not Cdh1 is present (Floyd et al., 2008). The importance of the change in APC/C specificity in different phases of the cell cycle is illustrated by the genetic instability of cells lacking Cdh1 (García-Higuera et al., 2008; Sigl et al., 2009), and the observation that perturbing the destruction of late mitotic substrates leads to problems in cytokinesis (Floyd et al., 2008; Lindon and Pines, 2004).

Recent biochemical and structural data indicate that the APC/C recognises some of its substrates through a bi-partite receptor composed of co-activator (Cdc20 or Cdh1) and the APC10 subunit (Buschhorn et al., 2011; Chao et al., 2012; da Fonseca et al., 2011; Izawa and Pines, 2011; Matyskiela and Morgan, 2009; Passmore and Barford, 2005). Exactly how the substrate binds into this receptor is not yet clear, but the interactions between Cdc20 and two checkpoint proteins in the structure of the Mitotic Checkpoint Complex (Chao et al., 2012) indicate that a KEN-box can bind to the top surface of Cdc20. By contrast, the D-box appears to bind to the side of Cdc20, between blades 1 and 7 of the beta-propeller domain (Chao et al., 2012) and to the Doc domain of APC10 (Carroll et al., 2005; da Fonseca et al., 2011; Passmore et al., 2003).

Given the importance of the cell cycle-regulated destruction of the B-type cyclins, we set out to define better how cyclin B1 is recognised by the APC/C through the cell cycle. We have found that the destruction motif that is recognised in metaphase overlaps with, but is distinct from, that recognised in anaphase and G1 phase, and this change is not dependent on replacing Cdc20 with Cdh1. Furthermore, several residues important for destruction are dispensable if Cyclin B1 can be recruited to the APC/C in anaphase, but not in metaphase. We conclude that multiple motifs are required to mediate recognition by the APC/C and these change as cells progress through mitosis. This may contribute to the ability of the APC/C to recognise different proteins at different times in mitosis.

## RESULTS

### Leucine 45 is the most critical residue in the Cyclin B1 Destruction box

Previous analyses of the residues required for a functional D-box primarily measured protein half-lives and not the point in mitosis that the protein became unstable (Glotzer et al., 1991; King et al., 1996; Yamano et al., 1998). To assay the timing of destruction we set up a live-cell assay in which we linked wild type or mutant human cyclin B1 to a fluorescent protein (CFP or YFP) to enable us to compare their destruction in the same cell (in all cases we checked that the same results were obtained when we swapped the fluorescent tags). To analyse the destruction of proteins in metaphase, protein values were normalised to those at NEBD; to analyse destruction in anaphase data were normalised to the beginning of anaphase. Note that we obtained similar results in assays where we depleted endogenous cyclin B1 by siRNA (directed against the 3′ UTR that is not present in the transgene) to exclude differences in degradation timing caused by competition with the endogenous protein, and that in agreement with previous studies (Wolf et al., 2006), non-degradable cyclin B1 blocked cells in anaphase rather than in metaphase.

**Fig. 1.**
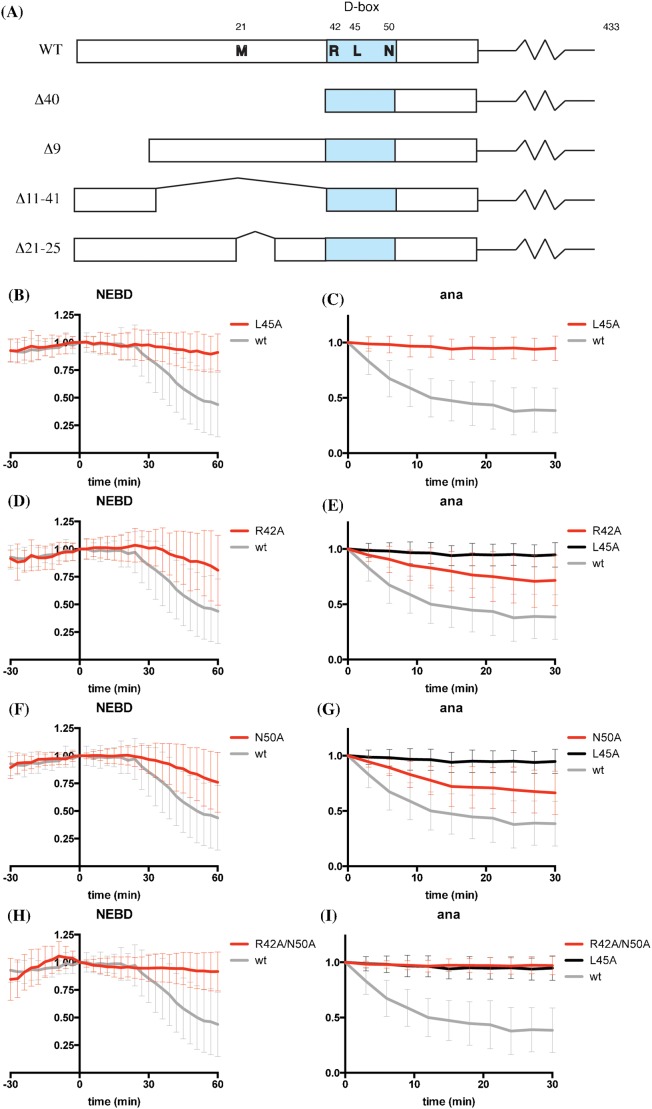
L45 of the Cyclin B1 D-box is most important for degradation. (A) Schematics of cyclin B1 constructs used in this study. The D-box is highlighted in blue. (B,C) HeLa cells were injected with cyclin B1-Venus (grey, n = 36) or cyclin B1 L45A-Venus (red, n = 24) constructs and followed by time-lapse fluorescence and DIC microscopy at 3-min intervals. The total fluorescence minus background was quantified for each cell in successive images of a time series and plotted over time as mean ± SD from 3 independent experiments. Fluorescence of cells at NEBD (B) or anaphase onset (C) was set to 1. Time 0 is NEBD in (B) or anaphase onset in (C). (D,E) HeLa cells were injected with cyclin B1-Venus (grey, n = 36), L45A-Venus (black, n = 24) or cyclin B1 R42A-Venus (red, n = 55) constructs and analysed as in panels B and C. Fluorescence of cells at NEBD (D) or anaphase onset (E) was set to 1. Time 0 is NEBD in (D) or anaphase onset in (E). Data are from 3 independent experiments. (F,G) HeLa cells were injected with cyclin B1-Venus (grey, n = 36), L45A-Venus (black, n = 24) or cyclin B1 N50A-Venus (red, n = 33) constructs and analysed as in panels B and C. Fluorescence of cells at NEBD (F) or anaphase onset (G) was set to 1. Time 0 is NEBD in (F) or anaphase onset in (G). Data are from 3 (wt and L45A) or 2 (N50A) independent experiments. (H,I) HeLa cells were injected with cyclin B1-Venus (grey, n = 36), L45A-Venus (black, n = 24) or cyclin B1 R42A/N50A-Venus (red, n = 38) constructs and analysed as in panels B and C. Data are from 3 independent experiments.

We first analysed the effect of mutating those conserved residues of the cyclin B1 D-box that had previously been shown to be most critical for destruction: R42, L45, and N50 (King et al., 1996; Yamano et al., 1998) (Fig. 1A). L45 proved to be essential for degradation in both metaphase and anaphase (Fig. 1B,C). The likely explanation for this is that the structure of the putative D-box binding site on Cdc20 indicated that L45 should be buried in a deep pocket (Chao et al., 2012). By contrast, we found mutating R42, which is commonly used to inactivate a D-box, only partially stabilised Cyclin B1 in anaphase (Fig. 1D,E). Mutating N50 had a similar effect to mutating R42 (Fig. 1F,G), but mutating both residues stabilised cyclin B1 in both metaphase and anaphase, in a similar fashion to mutating L45 (Fig. 1H,I).

### Identifying an Anaphase Degron

**Fig. 2.**
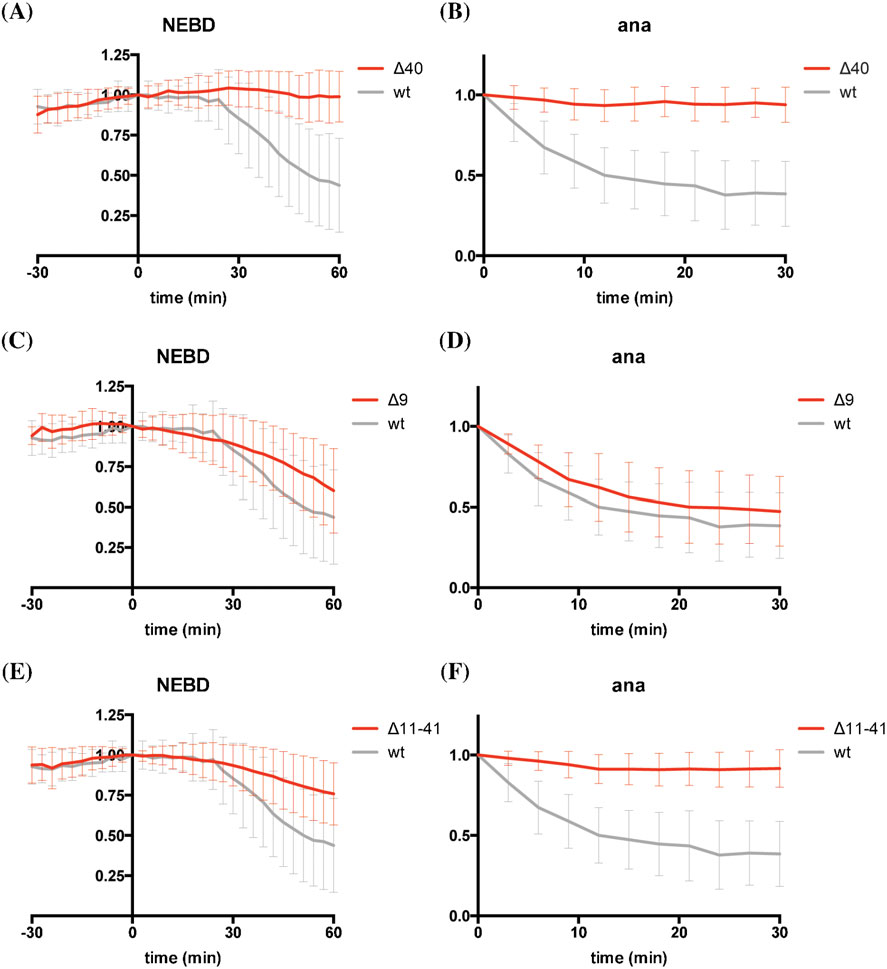
The first 40 amino acids of cyclin B1 are required for its degradation. (A,B) HeLa cells were injected with cyclin B1-Venus (grey, n = 36) or cyclin B1 Δ40-Venus (red, n = 45) constructs and analysed as in Fig. 1. Data are from 3 independent experiments. (C,D) HeLa cells were injected with cyclin B1-Venus (grey, n = 36) or cyclin B1 Δ9-Venus (red, n = 43) constructs and analysed as in Fig. 1. Data are from 3 independent experiments. (E,F) HeLa cells were injected with cyclin B1-Venus (grey, n = 36) or cyclin B1 Δ11–41-Venus (red, n = 47) constructs and analysed as in Fig. 1. Data are from 3 independent experiments. Note that the data for wild type cyclin B1 degradation are the same as those in Fig. 1.

Since the APC/C recognises a wider range of substrates in anaphase than in metaphase, we considered the possibility that different residues in cyclin B1 might be important for recognition in anaphase. Deleting the first 40 amino acids (i.e. very close to the start of the original D-box, Fig. 1A) blocked degradation in mitosis altogether (Fig. 2A,B), as previously observed for sea urchin cyclin B and for fission yeast securin/cut2 (King et al., 1996; Yamano et al., 1998). Removing the first 9 amino acids slightly slowed down metaphase degradation, but not anaphase degradation (Fig. 2C,D). An internal deletion from amino acids 11 to 41 also perturbed cyclin B1 degradation in metaphase, and appeared to have an even greater effect in anaphase (Fig. 2E,F).

**Fig. 3.**
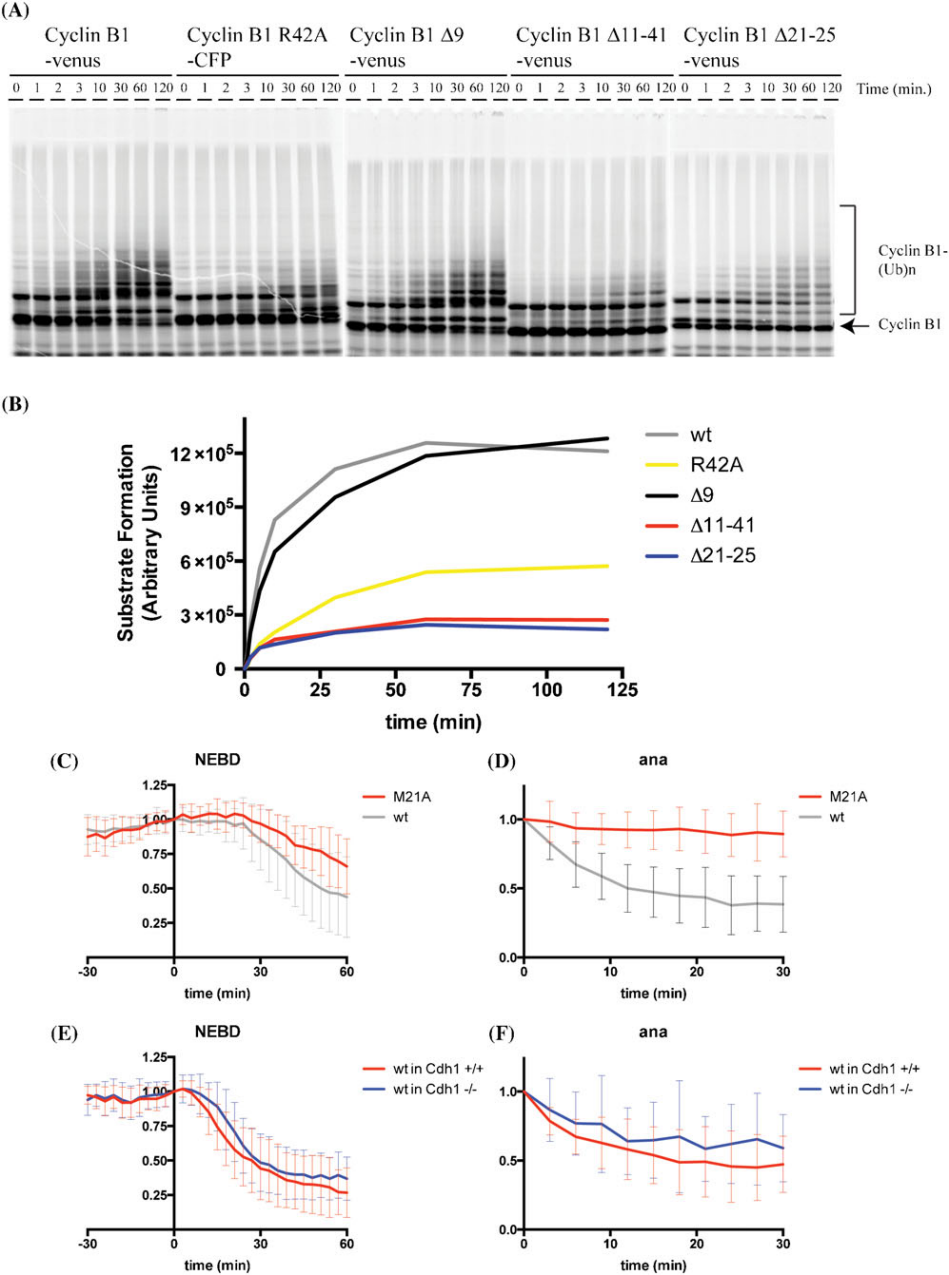
Residues N-terminal to the canonical D-box of cyclin B1 are required for its ubiquitylation and degradation in anaphase. (A) *In vitro* ubiquitylation reactions using purified budding yeast APC/C-Cdh1 and the indicated in vitro translated human cyclin B1 mutants. Data are representative of results from two independent experiments. (B) The data from (A) were quantified and the amount of ubiquitylated cyclin B1 was plotted as a function of time. (C,D) HeLa cells were injected with cyclin B1-Venus (grey, n = 36) or cyclin B1 M21A-Venus (red, n = 20) constructs and analysed as in Fig. 1. Data are from 3 independent experiments. (E,F) Cdh1^+/+^ (red), or Cdh1^−/−^ (blue) mouse embryonic fibroblasts were transfected with cyclin B1-Venus constructs and analysed as in Fig. 1. Error bars indicate mean ± SD of 41 and 50, cells, for panels E and F, respectively.

To narrow down the region responsible for promoting anaphase recognition by the APC/C we used an *in vitro* ubiquitylation system (Enquist-Newman et al., 2008) and found that the Δ11–41 mutant could hardly be ubiquitylated (Fig. 3A,B). We made a series of smaller internal deletions and found that deleting residues 21 to 25 greatly reduced ubiquitylation by APC/C *in vitro* (Fig. 3A,B). Moreover, this mutant was also stabilised in anaphase *in vivo* (supplementary material Fig. S1A,B). To refine this analysis we constructed point mutations in each of the residues from positions 21 to 25 and identified M21 as the critical residue for anaphase degradation (Fig. 3C,D). (Positions 22, 23, 24 are A–G–A, and mutating K25 to alanine did not affect degradation, data not shown.) Cells expressing the M21A mutant arrested in anaphase and it was notable that M21 was not required for degradation in metaphase (Fig. 3C).

What caused this change in APC/C specificity in anaphase? The obvious candidate was the exchange of Cdc20 for Cdh1, but we found there was no difference in the ability of mouse embryonic fibroblasts (MEFs) lacking Cdh1 (García-Higuera et al., 2008) to degrade cyclin B1 in metaphase or anaphase (Fig. 3E,F). Moreover, the requirement for M21 for degradation in anaphase was conserved (supplementary material Fig. S1C,D). We also excluded the possibility that the M21A mutant inhibited the anaphase APC/C because HeLa cells could still degrade the R42A mutant and Plk1 (supplementary material Fig. S1E). By contrast, Aurora A was stabilised as expected because the stabilised Cyclin B1-Cdk1 activity in anaphase prevented the activation of Cdh1 (Floyd et al., 2008). We conclude that the metaphase and anaphase APC/Cs recognise the same substrate in different ways: R42, N50 and particularly L45, are important for a functional D-box in metaphase, whereas in anaphase there is an additional requirement for M21.

### Multi-valent recruitment of Cyclin B1 to the APC/C is required for its degradation in metaphase and anaphase

**Fig. 4.**
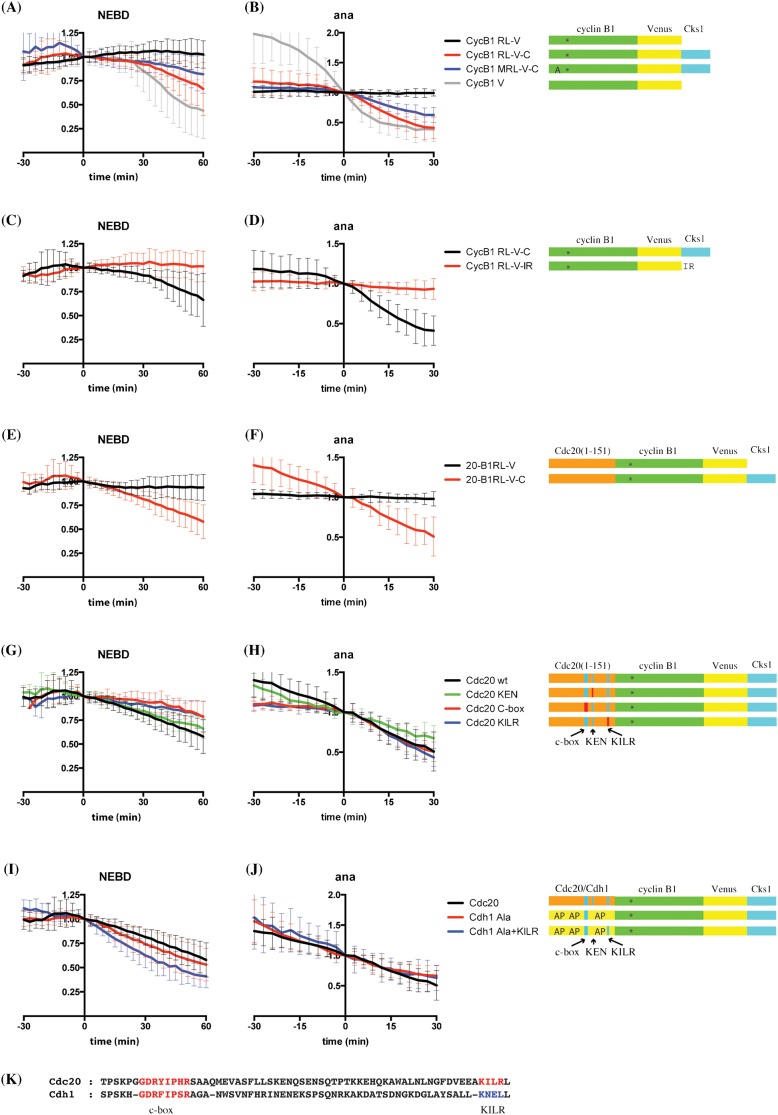
The N-terminus of Cdc20 can substitute for a D-box when cyclin B1 is targeted to the APC/C by Cks1. (A,B) The anaphase APC/C can degrade a D-box mutant of cyclin B1 targeted to it by Cks1. HeLa cells were injected with cyclin B1 R42A/L45A double mutant-venus (RL-V, black, n = 35), cyclin B1 R42A/L45A double mutant-venus-cks1 (RL-V-C, red, n = 24), cyclin B1 M21A/R42A/L45A triple mutant venus-cks1 (MRL-V-C, blue, n = 20), or cyclin B1-venus (grey, n = 36) constructs and analysed as in Fig. 1. Data are from 3 independent experiments. (C,D) Cks1 cannot be substituted by a C-terminal IR motif. HeLa cells were injected with cyclin B1 R42A/L45A double mutant-venus-cks1 (RL-V-C, black, n = 24) or cyclin B1 R42A/L45A double mutant-venus-IR (RL-V-IR, red, n = 29) constructs and analysed as in Fig. 1. Data are from 3 independent experiments. (E,F) The N-terminus of Cdc20 can promote degradation of cyclin B1 lacking its destruction box in metaphase if it is targeted to the APC/C by Cks1. The N-terminal 151 residues of Cdc20 were fused to cyclin B1 lacking its D-box, designated Cdc20 (1–151)-R42A/L45A double mutant cyclin B1, with or without Cks1 at the C-terminus. HeLa cells were injected with Cdc20 (1–151) cyclin B1 R42A/L45A double mutant-venus (20-B1RL-V, black, n = 24) or Cdc20 (1–151) cyclin B1 R42A/L45A double mutant-venus-cks1 (20-B1RL-V-C, red, n = 31) constructs and analysed as in Fig. 1. Data are from 3 independent experiments. (G,H) The N-terminus of Cdc20 requires its C-box and KILR motif to promote degradation. HeLa cells were injected with Cdc20 (1–151) cyclin B1 R42A/L45A double mutant-venus-cks1 (Cdc20 wt, black, n = 31), Cdc20 (1–151) KEN cyclin B1 R42A/L45A double mutant-venus-cks1 (Cdc20 KEN, green, n = 14), Cdc20 (1–151) C-box cyclin B1 R42A/L45A double mutant-venus-cks1 (Cdc20 C-box, red, n = 14), or Cdc20 (1–151) KILR cyclin B1 R42A/L45A double mutant-venus-cks1 (Cdc20 KILR, blue, n = 14) constructs and analysed as in Fig. 1. Data are from 3 independent experiments. (I,J) The non-phosphorylatable mutant N-terminus of Cdh1 can also promote degradation of cyclin B1 lacking its D-box. HeLa cells were injected with plasmids encoding Cdc20 (1–151) cyclin B1 R42A/L45A double mutant-venus-cks1 (Cdc20, black, n = 31), or a construct in which the N-terminus of Cdc20 was replaced with the N-terminus of non-phosphorylatable Cdh1, Cdh1 (1–155) Ala-cyclin B1 R42A/L45A double mutant-venus-cks1 (Cdh1 Ala, red, n = 16), or the N-terminus of non-phosphorylatable Cdh1 with the KILR motif of Cdc20, Cdh1 (1–155) Ala+KILR-cyclin B1 R42A/L45A double mutant-venus-cks1 (Cdh1 Ala+KILR, blue, n = 27). Constructs were analysed as in Fig. 1. Data are from 3 independent experiments. (K) Sequence alignment of Cdc20 and Cdh1 around C-box and KILR motif. The residues marked in blue (KNEL) in Cdh1 were mutated to those found in Cdc20 (KILR).

The cyclin B1-Cdk1 complex has previously been shown to bind to the APC/C through its partner Cks1 protein, and this improves the efficiency of its destruction (van Zon et al., 2010). To test whether the difference in D-box residues recognised by metaphase and anaphase APC/Cs was due to a difference in their ability to bind cyclin B1, we recruited wild type or a R42A/L45A double mutant of cyclin B1 directly to the APC/C by fusing its carboxyl terminus to the Cks1 protein (Di Fiore and Pines, 2010; Wolthuis et al., 2008). This revealed a marked difference between metaphase and anaphase cells: recruiting the double mutant to the metaphase APC/C had no effect, but recruiting it to the anaphase APC/C allowed it to be degraded (Fig. 4A,B). These experiments further underlined the importance of M21 to anaphase recognition because mutating M21 partially stabilised this construct in anaphase (Fig. 4A,B). Note that we could not substitute Cks1 with a C-terminal IR motif (Fig. 4C,D); therefore it may be important where and how the C-terminus of cyclin B1 binds to the APC/C.

Our results also indicated that APC/C appeared to have a more stringent requirement for the canonical D-box to recognise its substrates in metaphase than in anaphase. Therefore, we asked whether we could substitute the D-box of cyclin B1 with another APC/C-binding motif to confer metaphase destruction. We selected the N-terminus of Cdc20, which directed the degradation of Mes1 (Kimata et al., 2008). Kimata et al. found that the N-terminus of Cdc20 could not direct the degradation of cyclin B (Kimata et al., 2008), and we confirmed this result when we fused the N-terminus of Cdc20 to the R42A/L45A double mutant of cyclin B1 (Fig. 4E,F). Kimata et al. also showed that the N-terminus of Cdc20 could direct the destruction of Nek2A *in trans*. Nek2A differs from cyclin B1 in that it can bind directly to the APC/C through its IR tail; therefore, we tested whether binding cyclin B1 to the APC/C through Cks1 would now allow the N-terminus of Cdc20 to substitute for a D-box, and found that it did (Fig. 4E,F). Furthermore, this degradation did not require the KEN box (Fig. 4G,H) but did require the C-box and the KILR motif (Fig. 4G,H), the two known APC/C-binding motifs in the N-terminus of Cdc20. Similarly, a non-phosphorylatable version of the N-terminus of Cdh1 was also able to direct degradation of the R42A/L45A mutant fused to Cks1 (Fig. 4I,J), and was noticeably more potent in metaphase, but not anaphase, when we mutated its KILR motif to mimic that in Cdc20 (Fig. 4I–K).

## DISCUSSION

In this study we have investigated how the APC/C recognises its different substrates at different times by asking whether the key mitotic substrate, cyclin B1, is recognised in the same way in different mitotic phases. Cyclin B1 is first recognised in metaphase when the SAC is inactivated (Clute and Pines, 1999) but continues to be degraded in anaphase because cells enter anaphase with substantial amounts of cyclin B1 remaining (Collin et al., 2013). It is essential to degrade the remaining cyclin B1 for cells to undergo cytokinesis and exit mitosis (Wolf et al., 2006), and thus cells expressing the M21A mutant arrest in anaphase. We were surprised to find that the APC/C recognises overlapping but distinct motifs on cyclin B1 depending on whether it was in metaphase or anaphase: in metaphase the canonical D-box is most important, whereas in anaphase an additional region 20 amino acids N-terminal to the D-box is also important. In particular we identified M21 as crucial for anaphase degradation, and this residue is conserved in mammalian B1 cyclins but not in other vertebrates. Furthermore, the M21 region is important in anaphase cells that lack the Cdh1 co-activator protein, indicating that the change in APC/C substrate recognition must involve processes other than simply exchanging Cdc20 for Cdh1. We interpret these results as showing that the substrate interaction surfaces on the APC/C differ in a cell cycle-dependent manner.

At present we do not have a molecular mechanism for what causes the change in substrate recognition. Aside from the exchange of Cdc20 for Cdh1, the most prominent change in an anaphase cell compared to a metaphase cells is the change in the balance of protein kinases and phosphatases that is required for cytokinesis (Cundell et al., 2013); therefore, there may be a change in the phosphorylation state of substrate interaction motifs on the APC/C, although we find that any change does not require the inactivation of cyclin B1-cdk1. Alternatively, the region around M21 might be important for other aspects of APC/C-dependent ubiquitylation such as the addition of ubiquitin chains, or the pattern of the added chains that could alter the affinity for the proteasome. It is also conceivable that the change in substrate recognition is due to a change in the E2 used in anaphase, and indeed we previously found that UbcH10 begins to be degraded in anaphase (Walker et al., 2008).

The difference in the importance of the D-box for recognition by the APC/C in metaphase and anaphase is also illustrated by our finding that it can be dispensed with completely in anaphase if a substrate is recruited directly to the APC/C through the Cks1 protein, which binds to phosphorylated APC/C (Rudner and Murray, 2000). By comparison, this is not sufficient to degrade a substrate in metaphase. Both biochemical and structural data (Carroll et al., 2005; da Fonseca et al., 2011; Kraft et al., 2005; Passmore and Barford, 2005) indicate that the D-box is recognised by a bi-partite receptor composed of the co-activator and the APC10 subunit, but our data indicate that there may be differences between the metaphase and anaphase APC/Cs in substrate recognition surfaces outside this receptor.

We are able to destabilise cyclin B1 in metaphase when we recruit it to the APC/C through Cks1 and replace its D-box with another APC/C binding motif: the N-terminus of Cdc20. This adds to the accumulating evidence that substrate binding to the APC/C requires the cooperation of multiple interaction motifs (Burton and Solomon, 2001; Hames et al., 2001; Sedgwick et al., 2013; Wolthuis et al., 2008). Binding through multiple cooperating motifs rather than one high affinity interaction site would offer the opportunity for the APC/C to evolve interaction surfaces to favour the recognition of multiple substrates at different times in the cell cycle.

## MATERIALS AND METHODS

### Cell culture and synchronization

HeLa cells were cultured in Advanced DMEM (Life Technologies Ltd, Paisley, UK) supplemented with 2% foetal bovine serum, glutamax-I (200 µM), penicillin (100 U/ml), streptomycin (100 µg/ml) and fungizone (250 ng/ml) at 37°C, 10% CO_2_. MEFs were cultured in the medium previously described (García-Higuera et al., 2008). HeLa cells were synchronised at G1/S transition by a thymidine/aphidicolin block: the day after seeding, cells were blocked with thymidine (2.5 mM, Sigma–Aldrich, Gillingham, UK) for 24 hours, released for 12 hours and then blocked again with aphidicolin (2.5 µg/ml, Sigma–Aldrich) for 24 hours. Cells were then released in fresh DMEM.

### Microinjection and time-lapse imaging and analysis

For microinjection and microscopy, the cells were grown on a Bioptechs ΔT heating stage (Bioptechs, Butler, PA) attached to a Leica DMIRBE microscope and the culture medium was replaced with Leibovitz's L-15 medium (Life Technologies Ltd) supplemented with 10% foetal bovine serum, penicillin (100 U/ml) and streptomycin (100 µg/ml). Cells were microinjected with cDNA encoding cyclin B1-Venus at a concentration of 3 ng/µl in G2 cells, using a semiautomatic microinjector (Eppendorf, Stevenage, UK) on a Leica DMIRBE microscope (Leica Microsystems, Milton Keynes, UK) and assayed by time-lapse DIC and fluorescence microscopy as previously described (Karlsson and Pines, 1998). Parameters used for all images captured were exposure time 200 mseconds, 40× oil objective lens with a numerical aperture of 1.2. All images were captured at 3 minutes intervals and analyzed by SlideBook software (Intelligent Imaging Innovations, Denver, CO, USA).

### Ubiquitylation assay

E1, E2, Cdh1, and APC/C were expressed and purified as described previously (Carroll et al., 2005; Enquist-Newman et al., 2008). Substrates were transcribed and translated *in vitro* using the TNT system (Promega, Madison, WI) from plasmids with ^35^S-methionine and treated with 10 mM NEM (10 minutes) followed by 20 mM DTT (10 minutes) to inactivate ubiquitin chain-extending activities in the reticulocyte lysate. E1 (Uba1, 300 nM), E2 (Ubc4, 50 µM), ubiquitin (150 µM), and ATP (1 mM) were incubated for 15 minutes. APC/C (0.1–1 nM), substrate (2 µl of TnT mix into 15 µl reaction), and Cdh1 (2 µl of TnT mix into 15 µl reaction) were added. Reaction were incubated for the indicated times at room temperature, stopped by the addition of SDS sample buffer, separated by SDS-PAGE, and visualized and quantified with a Molecular Dynamics Phosphorimager (GE Healthcare, Fairfield, CT).

## Supplementary Material

Supplementary Material

## References

[b1] Amon A. Irniger S. Nasmyth K. (1994). Closing the cell cycle circle in yeast: G2 cyclin proteolysis initiated at mitosis persists until the activation of G1 cyclins in the next cycle. Cell 77, 1037–1050 10.1016/0092-8674(94)90443-X 8020094

[b2] Araki M. Wharton R. P. Tang Z. Yu H. Asano M. (2003). Degradation of origin recognition complex large subunit by the anaphase-promoting complex in Drosophila. EMBO J. 22, 6115–6126 10.1093/emboj/cdg573 14609957 PMC275432

[b3] Barford D. (2011). Structural insights into anaphase-promoting complex function and mechanism. Philos. Trans. R. Soc. B 366, 3605–3624 10.1098/rstb.2011.0069 PMC3203452 22084387

[b4] Brandeis M. Hunt T. (1996). The proteolysis of mitotic cyclins in mammalian cells persists from the end of mitosis until the onset of S phase. EMBO J. 15, 5280–5289. 8895573 PMC452272

[b5] Burton J. L. Solomon M. J. (2001). D box and KEN box motifs in budding yeast Hsl1p are required for APC-mediated degradation and direct binding to Cdc20p and Cdh1p. Genes Dev. 15, 2381–2395 10.1101/gad.917901 11562348 PMC312781

[b6] Buschhorn B. A. Petzold G. Galova M. Dube P. Kraft C. Herzog F. Stark H. Peters J. M. (2011). Substrate binding on the APC/C occurs between the coactivator Cdh1 and the processivity factor Doc1. Nat. Struct. Mol. Biol. 18, 6–13 10.1038/nsmb.1979 21186364 PMC4300845

[b7] Carroll C. W. Enquist-Newman M. Morgan D. O. (2005). The APC subunit Doc1 promotes recognition of the substrate destruction box. Curr. Biol. 15, 11–18 10.1016/j.cub.2004.12.066 15649358

[b8] Castro A. Vigneron S. Bernis C. Labbé J. C. Lorca T. (2003). Xkid is degraded in a D-box, KEN-box, and A-box-independent pathway. Mol. Cell. Biol. 23, 4126–4138 10.1128/MCB.23.12.4126-4138.2003 12773557 PMC156142

[b9] Chao W. C. Kulkarni K. Zhang Z. Kong E. H. Barford D. (2012). Structure of the mitotic checkpoint complex. Nature 484, 208–213 10.1038/nature10896 22437499

[b10] Clute P. Pines J. (1999). Temporal and spatial control of cyclin B1 destruction in metaphase. Nat. Cell Biol. 1, 82–87 10.1038/10049 10559878

[b11] Collin P. Nashchekina O. Walker R. Pines J. (2013). The spindle assembly checkpoint works like a rheostat rather than a toggle switch. Nat. Cell Biol. 15, 1378–1385 10.1038/ncb2855 24096242 PMC3836401

[b12] Cundell M. J. Bastos R. N. Zhang T. Holder J. Gruneberg U. Novák B. Barr F. A. (2013). The BEG (PP2A-B55/ENSA/Greatwall) pathway ensures cytokinesis follows chromosome separation. Mol. Cell 52, 393–405 10.1016/j.molcel.2013.09.005 24120663 PMC3898901

[b13] da Fonseca P. C. A. Kong E. H. Zhang Z. Schreiber A. Williams M. A. Morris E. P. Barford D. (2011). Structures of APC/C(Cdh1) with substrates identify Cdh1 and Apc10 as the D-box co-receptor. Nature 470, 274–278 10.1038/nature09625 21107322 PMC3037847

[b14] den Elzen N. Pines J. (2001). Cyclin A is destroyed in prometaphase and can delay chromosome alignment and anaphase. J. Cell Biol. 153, 121–136 10.1083/jcb.153.1.121 11285279 PMC2185531

[b15] Di Fiore B. Pines J. (2010). How cyclin A destruction escapes the spindle assembly checkpoint. J. Cell Biol. 190, 501–509 10.1083/jcb.201001083 20733051 PMC2928024

[b16] Enquist-Newman M. Sullivan M. Morgan D. O. (2008). Modulation of the mitotic regulatory network by APC-dependent destruction of the Cdh1 inhibitor Acm1. Mol. Cell 30, 437–446 10.1016/j.molcel.2008.04.004 18498748 PMC2494983

[b17] Fang G. Yu H. Kirschner M. W. (1998). The checkpoint protein MAD2 and the mitotic regulator CDC20 form a ternary complex with the anaphase-promoting complex to control anaphase initiation. Genes Dev. 12, 1871–1883 10.1101/gad.12.12.1871 9637688 PMC316912

[b18] Floyd S. Pines J. Lindon C. (2008). APC/C Cdh1 targets aurora kinase to control reorganization of the mitotic spindle at anaphase. Curr. Biol. 18, 1649–1658 10.1016/j.cub.2008.09.058 18976910

[b19] García-Higuera I. Manchado E. Dubus P. Cañamero M. Méndez J. Moreno S. Malumbres M. (2008). Genomic stability and tumour suppression by the APC/C cofactor Cdh1. Nat. Cell Biol. 10, 802–811 10.1038/ncb1742 18552834

[b20] Geley S. Kramer E. Gieffers C. Gannon J. Peters J. M. Hunt T. (2001). Anaphase-promoting complex/cyclosome-dependent proteolysis of human cyclin A starts at the beginning of mitosis and is not subject to the spindle assembly checkpoint. J. Cell Biol. 153, 137–148 10.1083/jcb.153.1.137 11285280 PMC2185534

[b21] Glotzer M. Murray A. W. Kirschner M. W. (1991). Cyclin is degraded by the ubiquitin pathway. Nature 349, 132–138 10.1038/349132a0 1846030

[b22] Hagting A. Den Elzen N. Vodermaier H. C. Waizenegger I. C. Peters J.-M. Pines J. (2002). Human securin proteolysis is controlled by the spindle checkpoint and reveals when the APC/C switches from activation by Cdc20 to Cdh1. J. Cell Biol. 157, 1125–1137 10.1083/jcb.200111001 12070128 PMC2173548

[b23] Hames R. S. Wattam S. L. Yamano H. Bacchieri R. Fry A. M. (2001). APC/C-mediated destruction of the centrosomal kinase Nek2A occurs in early mitosis and depends upon a cyclin A-type D-box. EMBO J. 20, 7117–7127 10.1093/emboj/20.24.7117 11742988 PMC125337

[b24] Hayes M. J. Kimata Y. Wattam S. L. Lindon C. Mao G. Yamano H. Fry A. M. (2006). Early mitotic degradation of Nek2A depends on Cdc20-independent interaction with the APC/C. Nat. Cell Biol. 8, 607–614 10.1038/ncb1410 16648845

[b25] Hoyt M. A. Totis L. Roberts B. T. (1991). S. cerevisiae genes required for cell cycle arrest in response to loss of microtubule function. Cell 66, 507–517 10.1016/0092-8674(81)90014-3 1651171

[b26] Hwang L. H. Lau L. F. Smith D. L. Mistrot C. A. Hardwick K. G. Hwang E. S. Amon A. Murray A. W. (1998). Budding yeast Cdc20: a target of the spindle checkpoint. Science 279, 1041–1044 10.1126/science.279.5353.1041 9461437

[b27] Izawa D. Pines J. (2011). How APC/C-Cdc20 changes its substrate specificity in mitosis. Nat. Cell Biol. 13, 223–233 10.1038/ncb2165 21336306 PMC3059483

[b28] Karlsson C. Pines J. (1998). Green fluorescent protein. Cell Biology: A Laboratory Handbook Celis J, ed246–252 San Diego, CA: Academic Press.

[b29] Kim S. H. Lin D. P. Matsumoto S. Kitazono A. Matsumoto T. (1998). Fission yeast Slp1: an effector of the Mad2-dependent spindle checkpoint. Science 279, 1045–1047 10.1126/science.279.5353.1045 9461438

[b30] Kimata Y. Baxter J. E. Fry A. M. Yamano H. (2008). A role for the Fizzy/Cdc20 family of proteins in activation of the APC/C distinct from substrate recruitment. Mol. Cell 32, 576–583 10.1016/j.molcel.2008.09.023 19026787

[b31] King R. W. Peters J. M. Tugendreich S. Rolfe M. Hieter P. Kirschner M. W. (1995). A 20S complex containing CDC27 and CDC16 catalyzes the mitosis-specific conjugation of ubiquitin to cyclin B. Cell 81, 279–288 10.1016/0092-8674(95)90338-0 7736580

[b32] King R. W. Glotzer M. Kirschner M. W. (1996). Mutagenic analysis of the destruction signal of mitotic cyclins and structural characterization of ubiquitinated intermediates. Mol. Biol. Cell 7, 1343–1357 10.1091/mbc.7.9.1343 8885231 PMC275986

[b33] Kraft C. Vodermaier H. C. Maurer-Stroh S. Eisenhaber F. Peters J. M. (2005). The WD40 propeller domain of Cdh1 functions as a destruction box receptor for APC/C substrates. Mol. Cell 18, 543–553 10.1016/j.molcel.2005.04.023 15916961

[b34] Leismann O. Herzig A. Heidmann S. Lehner C. F. (2000). Degradation of Drosophila PIM regulates sister chromatid separation during mitosis. Genes Dev. 14, 2192–2205 10.1101/gad.176700 10970883 PMC316890

[b35] Li R. Murray A. W. (1991). Feedback control of mitosis in budding yeast. Cell 66, 519–531 10.1016/0092-8674(81)90015-5 1651172

[b36] Lindon C. Pines J. (2004). Ordered proteolysis in anaphase inactivates Plk1 to contribute to proper mitotic exit in human cells. J. Cell Biol. 164, 233–241 10.1083/jcb.200309035 14734534 PMC2172335

[b37] Littlepage L. E. Ruderman J. V. (2002). Identification of a new APC/C recognition domain, the A box, which is required for the Cdh1-dependent destruction of the kinase Aurora-A during mitotic exit. Genes Dev. 16, 2274–2285 10.1101/gad.1007302 12208850 PMC186670

[b38] Matyskiela M. E. Morgan D. O. (2009). Analysis of activator-binding sites on the APC/C supports a cooperative substrate-binding mechanism. Mol. Cell 34, 68–80 10.1016/j.molcel.2009.02.027 19362536 PMC2754851

[b39] Morgan D. O. (2007). The Cell Cycle: Principles of Control Oxford: Oxford University Press.

[b40] Murray A. W. (2011). A brief history of error. Nat. Cell Biol. 13, 1178–1182 10.1038/ncb2348 21968991

[b41] Murray A. W. Solomon M. J. Kirschner M. W. (1989). The role of cyclin synthesis and degradation in the control of maturation promoting factor activity. Nature 339, 280–286 10.1038/339280a0 2566918

[b42] Passmore L. A. Barford D. (2005). Coactivator functions in a stoichiometric complex with anaphase-promoting complex/cyclosome to mediate substrate recognition. EMBO Rep. 6, 873–878 10.1038/sj.embor.7400482 16113654 PMC1369160

[b43] Passmore L. A. McCormack E. A. Au S. W. Paul A. Willison K. R. Harper J. W. Barford D. (2003). Doc1 mediates the activity of the anaphase-promoting complex by contributing to substrate recognition. EMBO J. 22, 786–796 10.1093/emboj/cdg084 12574115 PMC145444

[b44] Pfleger C. M. Lee E. Kirschner M. W. (2001). Substrate recognition by the Cdc20 and Cdh1 components of the anaphase-promoting complex. Genes Dev. 15, 2396–2407 10.1101/gad.918201 11562349 PMC312782

[b45] Pines J. (2006). Mitosis: a matter of getting rid of the right protein at the right time. Trends Cell Biol. 16, 55–63 10.1016/j.tcb.2005.11.006 16337124

[b46] Pines J. (2011). Cubism and the cell cycle: the many faces of the APC/C. Nat. Rev. Mol. Cell Biol. 12, 427–438 10.1038/nrm3132 21633387

[b47] Rape M. Reddy S. K. Kirschner M. W. (2006). The processivity of multiubiquitination by the APC determines the order of substrate degradation. Cell 124, 89–103 10.1016/j.cell.2005.10.032 16413484

[b48] Rieder C. L. Cole R. W. Khodjakov A. Sluder G. (1995). The checkpoint delaying anaphase in response to chromosome monoorientation is mediated by an inhibitory signal produced by unattached kinetochores. J. Cell Biol. 130, 941–948 10.1083/jcb.130.4.941 7642709 PMC2199954

[b49] Rudner A. D. Murray A. W. (2000). Phosphorylation by Cdc28 activates the Cdc20-dependent activity of the anaphase-promoting complex. J. Cell Biol. 149, 1377–1390 10.1083/jcb.149.7.1377 10871279 PMC2175139

[b50] Sedgwick G. G. Hayward D. G. Di Fiore B. Pardo M. Yu L. Pines J. Nilsson J. (2013). Mechanisms controlling the temporal degradation of Nek2A and Kif18A by the APC/C-Cdc20 complex. EMBO J. 32, 303–314 10.1038/emboj.2012.335 23288039 PMC3553385

[b51] Sigl R. Wandke C. Rauch V. Kirk J. Hunt T. Geley S. (2009). Loss of the mammalian APC/C activator FZR1 shortens G1 and lengthens S phase but has little effect on exit from mitosis. J. Cell Sci. 122, 4208–4217 10.1242/jcs.054197 19861496

[b52] Sudakin V. Ganoth D. Dahan A. Heller H. Hershko J. Luca F. C. Ruderman J. V. Hershko A. (1995). The cyclosome, a large complex containing cyclin-selective ubiquitin ligase activity, targets cyclins for destruction at the end of mitosis. Mol. Biol. Cell 6, 185–197 10.1091/mbc.6.2.185 7787245 PMC275828

[b53] Sudakin V. Chan G. K. Yen T. J. (2001). Checkpoint inhibition of the APC/C in HeLa cells is mediated by a complex of BUBR1, BUB3, CDC20, and MAD2. J. Cell Biol. 154, 925–936 10.1083/jcb.200102093 11535616 PMC2196190

[b54] van Zon W. Ogink J. ter Riet B. Medema R. H. te Riele H. Wolthuis R. M. (2010). The APC/C recruits cyclin B1-Cdk1-Cks in prometaphase before D box recognition to control mitotic exit. J. Cell Biol. 190, 587–602 10.1083/jcb.200912084 20733055 PMC2928021

[b55] Visintin R. Prinz S. Amon A. (1997). CDC20 and CDH1: a family of substrate-specific activators of APC-dependent proteolysis. Science 278, 460–463 10.1126/science.278.5337.460 9334304

[b56] Walker A. Acquaviva C. Matsusaka T. Koop L. Pines J. (2008). UbcH10 has a rate-limiting role in G1 phase but might not act in the spindle checkpoint or as part of an autonomous oscillator. J. Cell Sci. 121, 2319–2326 10.1242/jcs.031591 18559889

[b57] Wolf F. Wandke C. Isenberg N. Geley S. (2006). Dose-dependent effects of stable cyclin B1 on progression through mitosis in human cells. EMBO J. 25, 2802–2813 10.1038/sj.emboj.7601163 16724106 PMC1500859

[b58] Wolthuis R. Clay-Farrace L. van Zon W. Yekezare M. Koop L. Ogink J. Medema R. Pines J. (2008). Cdc20 and Cks direct the spindle checkpoint-independent destruction of cyclin A. *Mol.* Cell 30, 290–302 10.1016/j.molcel.2008.02.027 18471975

[b59] Yamano H. Tsurumi C. Gannon J. Hunt T. (1998). The role of the destruction box and its neighbouring lysine residues in cyclin B for anaphase ubiquitin-dependent proteolysis in fission yeast: defining the D-box receptor. EMBO J. 17, 5670–5678 10.1093/emboj/17.19.5670 9755167 PMC1170895

[b60] Yamashita Y. M. Nakaseko Y. Samejima I. Kumada K. Yamada H. Michaelson D. Yanagida M. (1996). 20S cyclosome complex formation and proteolytic activity inhibited by the cAMP/PKA pathway. Nature 384, 276–279 10.1038/384276a0 8918880

[b61] Yu H. (2007). Cdc20: a WD40 activator for a cell cycle degradation machine. Mol. Cell 27, 3–16 10.1016/j.molcel.2007.06.009 17612486

[b62] Zachariae W. Shin T. H. Galova M. Obermaier B. Nasmyth K. (1996). Identification of subunits of the anaphase-promoting complex of Saccharomyces cerevisiae. Science 274, 1201–1204 10.1126/science.274.5290.1201 8895471

[b63] Zur A. Brandeis M. (2001). Securin degradation is mediated by fzy and fzr, and is required for complete chromatid separation but not for cytokinesis. EMBO J. 20, 792–801 10.1093/emboj/20.4.792 11179223 PMC145417

[b64] Zur A. Brandeis M. (2002). Timing of APC/C substrate degradation is determined by fzy/fzr specificity of destruction boxes. EMBO J. 21, 4500–4510 10.1093/emboj/cdf452 12198152 PMC126191

