# Supplementary material for: Co-activator independent differences in how the metaphase and anaphase APC/C recognise the same substrate
Source: Biol Open. 2014 Sep 12;3(10):904–12. doi: 10.1242/bio.20149415 (PMC4197439; doi:10.1242/bio.20149415)
Supplement: Supplementary Material [file supp_3_10_904__index.html]

Co-activator independent differences in how the metaphase and anaphase APC/C recognise the same substrate — Co-activator independent differences in how the metaphase and anaphase APC/C recognise the same substrate — Supplementary Material 

# Co-activator independent differences in how the metaphase and anaphase APC/C recognise the same substrate

## bio.20149415 Supplementary Material

**Files in this Data Supplement:**

- Supplementary Material - Takahiro Matsusaka et al. doi: 10.1242/bio.20149415
