# Supplementary material for: Co-activator independent differences in how the metaphase and anaphase APC/C recognise the same substrate
Source: Biol Open. 2014 Sep 12;3(10):904–12. doi: 10.1242/bio.20149415 (PMC4197439; doi:10.1242/bio.20149415)
Supplement: Supplementary Material [file supp_bio.20149415_bio.20149415-s1.pdf]

Biology Open (2014) 000, 1–9 doi:10.1242/bio.20149415

Supplementary Material

Takahiro Matsusaka et al. doi: 10.1242/bio.20149415

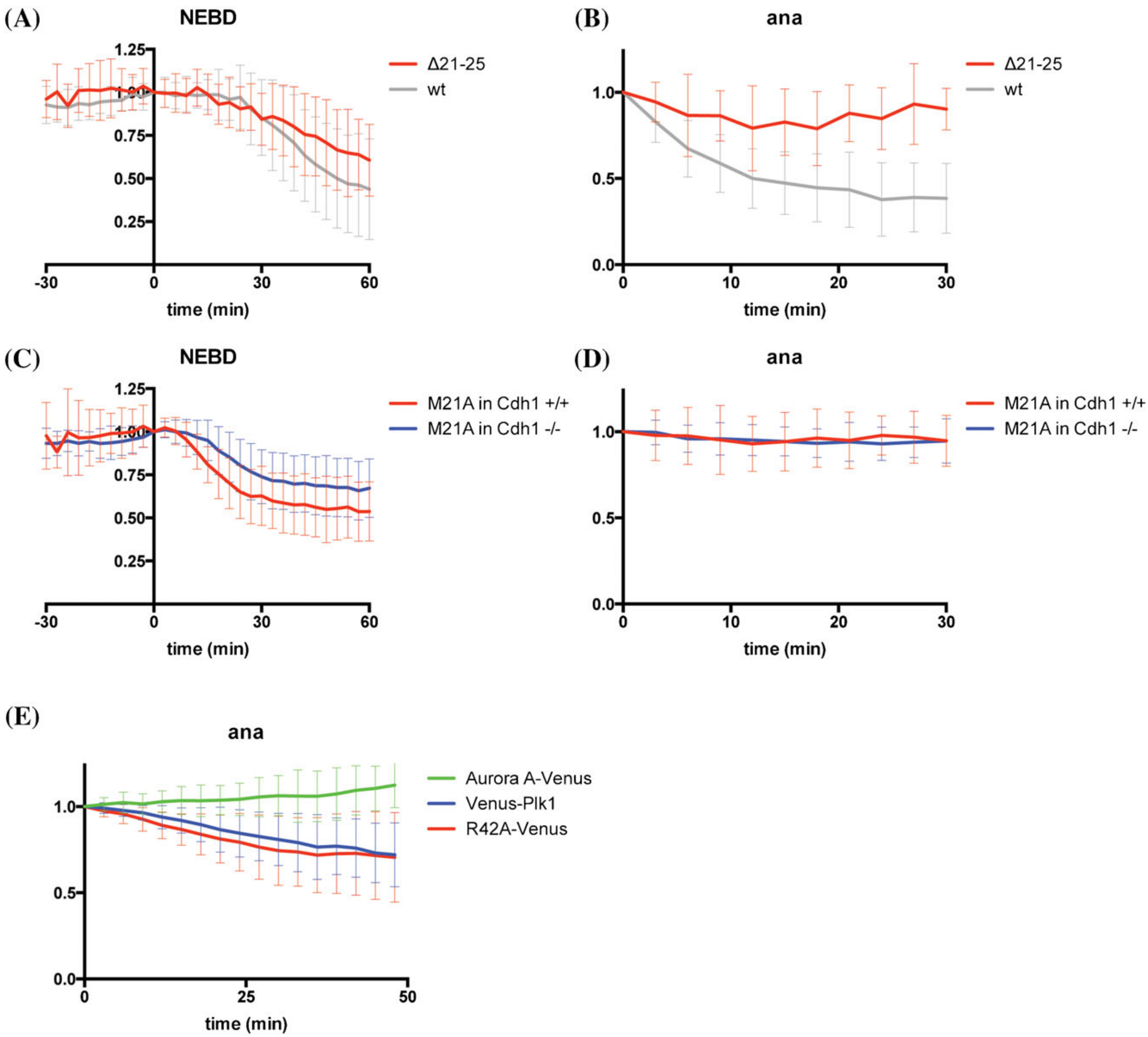

Fig. S1. Anaphase D-box recognition by the APC/C. (A,B) HeLa cells were injected with cyclin B1-Venus (grey, n=36) or cyclin B1 Δ21–25-Venus (red, n=25) constructs and analysed as in Fig. 1. Data are from 3 independent experiments. Note that the data for wild type cyclin B1 degradation are the same as those in Fig. 1. (C,D) Cdh1$^{+/+}$ (red), or Cdh1$^{-/-}$ (blue) mouse embryo fibroblasts were transfected with cyclin B1 M21A-Venus constructs and analysed as in Fig. 1. Error bars indicate mean ± SD of 32 and 41 cells for panels C and D, respectively. (E) HeLa cells were injected with cyclin B1-M21A-Cerulean and cyclin B1 R42AVenus (red, n=19), Venus-Plk1 (blue, n=13), or Aurora A-Venus (green, n=12) constructs and analysed as in Fig. 1. Data are from at least 2 independent experiments.
